# Cerebral Oxygen Saturation: Graded Response to Carbon Dioxide with Isoxia and Graded Response to Oxygen with Isocapnia

**DOI:** 10.1371/journal.pone.0057881

**Published:** 2013-02-28

**Authors:** W. Alan C. Mutch, Sunni R. Patel, Ayda M. Shahidi, Susith I. Kulasekara, Joseph A. Fisher, James Duffin, Christopher Hudson

**Affiliations:** 1 Department of Anesthesia and Perioperative Medicine, University of Manitoba, Winnipeg, Manitoba, Canada; 2 Department of Ophthalmology and Vision Sciences, University of Toronto, Toronto, Ontario, Canada; 3 Department of Anesthesia and Pain Management, University of Toronto, Toronto, Ontario, Canada; 4 Department of Physiology, University of Toronto, Toronto, Ontario, Canada; D’or Institute of Research and Education, Brazil

## Abstract

**Background:**

Monitoring cerebral saturation is increasingly seen as an aid to management of patients in the operating room and in neurocritical care. How best to manipulate cerebral saturation is not fully known. We examined cerebral saturation with graded changes in carbon dioxide tension while isoxic and with graded changes in oxygen tension while isocapnic.

**Methodology/Principal Findings:**

The study was approved by the Research Ethics Board of the University Health Network at the University of Toronto. Thirteen studies were undertaken in healthy adults with cerebral oximetry by near infrared spectroscopy. End-tidal gas concentrations were manipulated using a model-based prospective end-tidal targeting device. End-tidal carbon dioxide was altered ±15 mmHg from baseline in 5 mmHg increments with isoxia (clamped at 110±4 mmHg). End-tidal oxygen was changed to 300, 400, 500, 80, 60 and 50 mmHg under isocapnia (37±2 mmHg). Twelve studies were completed. The end-tidal carbon dioxide *versus* cerebral saturation fit a linear relationship (R^2^ = 0.92±0.06). The end-tidal oxygen *versus* cerebral saturation followed log-linear behaviour and best fit a hyperbolic relationship (R^2^ = 0.85±0.10). Cerebral saturation was maximized in isoxia at end-tidal carbon dioxide of baseline +15 mmHg (77±3 percent). Cerebral saturation was minimal in isocapnia at an end-tidal oxygen tension of 50 mmHg (61±3 percent). The cerebral saturation during normoxic hypocapnia was equivalent to normocapnic hypoxia of 60 mmHg.

**Conclusions/Significance:**

Hypocapnia reduces cerebral saturation to an extent equivalent to moderate hypoxia.

## Introduction

Regulation of end-tidal gas concentrations is critical for the management of patients undergoing neurosurgical procedures and in the neurocritical care setting to ensure adequate cerebral oxygenation and regulation of intracranial pressure and volume. [1]–[2] The monitoring of cerebral saturation by near infrared spectroscopy (NIRS) is contributing increasingly to the management of patients in these circumstances. [3] The response of the human brain to graded changes in end-tidal oxygen under isocapnic conditions, and graded changes in end-tidal carbon dioxide tensions under isoxic conditions, is however, poorly documented. A better understanding of the changes in cerebral saturation under such controlled experimental conditions can potentially benefit clinicians to aid in optimizing cerebral saturation in patients when cerebral hypoxia is present. It is well understood that hypocapnia can lower cerebral oxygenation via its vasoconstrictive effects and the converse occurs with hypercapnia. [2] [4]–[5] Brain bulk is also influenced by alterations in cerebral blood flow and volume seen with changes in carbon dioxide tension. [6] Also important is how graded changes in oxygen tension in normocapnia influence cerebral saturation. [7]–[8] Optimal regulation of end-tidal gas concentrations could dictate ventilation parameters where cerebral perfusion may be of concern; in the intensive care for patients with such conditions as traumatic brain injury and subarachnoid hemorrhage, and in the operating room during neurosurgery and open-heart surgery.

The means to independently control end-tidal carbon dioxide and oxygen tensions has recently become available. [9] Such an approach is called model-based prospective end-tidal targeting (MPET) and utilizes a computer-controlled gas blender with a sequential breathing circuit. [10] As well, newer cerebral oximeters are available which are deemed to provide absolute measures of cerebral saturation. [11] The combination of these two newer devices permits a comprehensive investigation of the effect of graded changes in oxygen and carbon dioxide on cerebral saturation. We have therefore undertaken such a study in healthy volunteers.

## Methods

The protocol was approved by the Research Ethics Board of the University Health Network at the University of Toronto. All subjects gave witnessed, written informed consent. All subjects denied neurological disease, including migraine headache. All were asked to refrain from drinking caffeinated beverages before their study. After an explanation of the nature of the study, the subjects were monitored by pulse oximetry, intermittent oscillometry to determine blood pressure, and had bifrontal sensors placed to monitor cerebral saturation. The subjects had a facemask secured about their mouth and nose in an airtight manner by the application of Tegaderm™ (3 M, St. Paul, MN) adhesive tape. The facemask was attached to a sequential breathing circuit (Thornhill Research Inc., Toronto, Canada). After a period of stabilization on, and acclimatization to the breathing circuit, the study began.

### Cerebral Oximetry

All subjects were monitored using the Fore-Sight™ cerebral oximeter (CasMed, Branford, CT). This device uses sensors applied bilaterally to the forehead to measure cerebral oxygen saturation by NIRS. The sensor optodes are supplied with four separate laser light wavelengths by the monitor. Output from the device is deemed to provide an absolute measure of cerebral saturation based on a ratio of 30% arterial saturation and 70% venous saturation. Each study period for the chosen end-tidal gas concentrations was time and date stamped at the beginning and end of the sample period. Data from the monitor were saved continuously at a 2-second sampling rate. At the end of the study these data were downloaded to an Excel spreadsheet for processing.

### Model-based Prospective End-Tidal Targeting (MPET) of Breathing Gas Mixtures

End-tidal carbon dioxide and oxygen were controlled using an MPET system (RespirAct™; Thornhill Research Inc., Toronto, Canada). The theory behind the device has been well described previously. [9] In short, by varying the end-tidal gas concentrations with a computer-controlled gas blender in concert with a custom designed sequential breathing circuit, end-tidal carbon dioxide and oxygen tensions can be independently controlled in a precise fashion. Cerebral oxygen saturation was assessed initially by decreasing the end-tidal carbon dioxide tension from baseline in 5 mmHg decrements to a total of 15 mmHg below baseline, and followed by 5 mmHg incremental increases in carbon dioxide tension up to 15 mmHg above baseline (Stage 1). In each circumstance the end-tidal concentration of oxygen was clamped at the baseline values recorded breathing medical air. At each target the end-tidal carbon dioxide tension was clamped to within 1–2 mmHg of the desired value. Isoxia was maintained within 2–5 mmHg of the baseline values. Cerebral oximetry data were acquired at the target end-tidal carbon dioxide tension when these readings were stable for longer than 60 seconds. A minimum of 2-minutes of data were obtained at each target.

Cerebral saturation values were allowed to re-equilibrate to baseline values before commencing the graded end-tidal oxygen study (Stage 2). Changes in end-tidal oxygen tensions were undertaken while maintaining a stable isocapnic baseline. End-tidal oxygen tensions were increased from the baseline value to 300, 400 and 500 mmHg. For each measurement period the target end-tidal oxygen and carbon dioxide tensions were clamped to within 1–2 mmHg of their desired values. Cerebral oximetry data were acquired when stable for longer than 60 seconds at the target end-tidal oxygen tension. A minimum of 2-minutes of data were obtained at each target. Following a period of re-equilibration to baseline after end-tidal oxygen tensions of 500 mmHg, end-tidal oxygen tensions were decreased to 80, 60 then 50 mmHg; again with end-tidal carbon dioxide tensions clamped at baseline values.

### Post-hoc Analysis

Data were downloaded from the pulse oximeter, blood pressure monitor, Fore-Sight monitor and RespirAct™ and entered into Excel spreadsheets to time align the data for each study period. Data were analyzed for each subject for all time periods and collated to examine the mean values. Correlations between end-tidal tensions of carbon dioxide and cerebral saturation and end-tidal tensions of oxygen and cerebral saturation were generated for each subject. Nonlinear curve fitting utilized a custom designed computer program (LabVIEW™, National Instruments, Austin, TX).

### Statistical Analysis

Linear and nonlinear regression analyses were undertaken. Repeated measures ANOVA was used to examine the changes in hemodynamics and end-tidal tensions of gases over time. A p-value of p<0.05 was considered statistically significant following correction for multiple comparisons (Tukey’s test). Specific within group comparisons were made by paired t-test; p<0.05 was considered significant.

## Results

**Figure 1:**
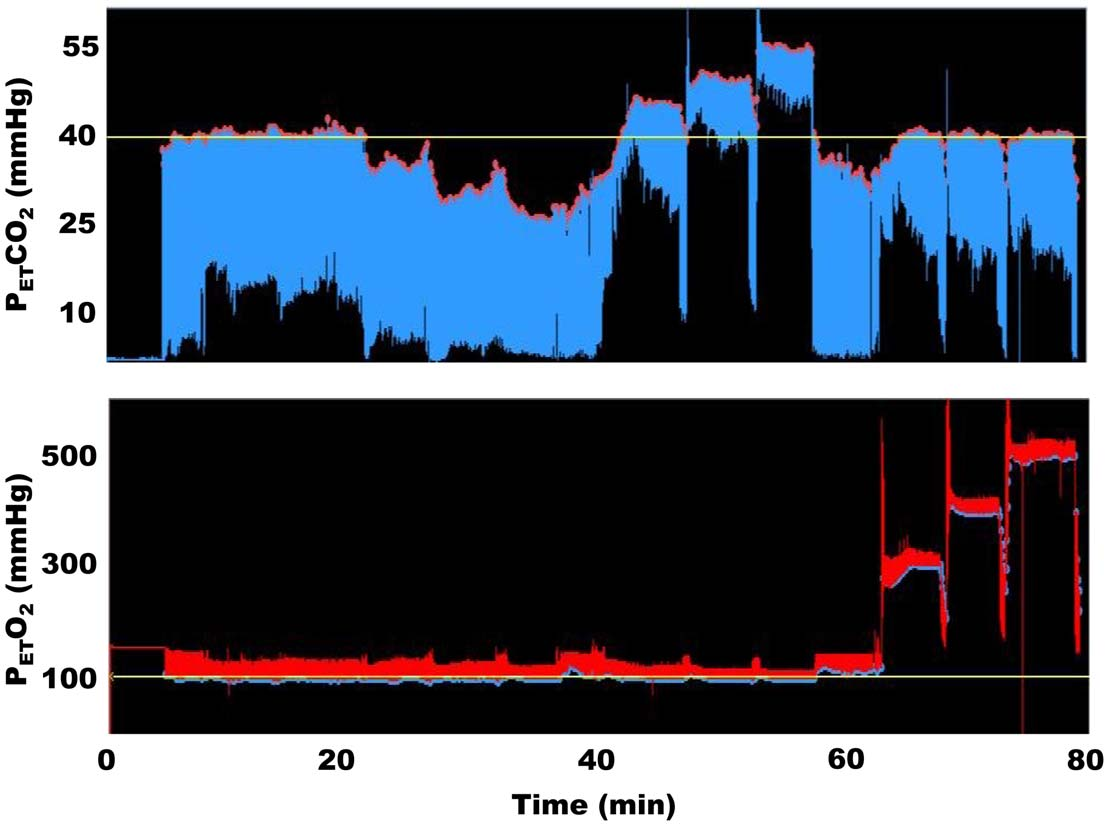
Model-based prospective end-tidal gas targeting (MPET) in one subject. The x-axis is labelled in minutes. Note stable end-tidal oxygen tensions during manipulation of end-tidal carbon dioxide and vice versa. For each sequence the breath-by-breath end-tidal carbon dioxide tensions are shown as the solid red dots on the top of the blue waveform trace and end-tidal oxygen tensions as the solid blue dots on the bottom of the red waveform trace.

**Figure 2:**
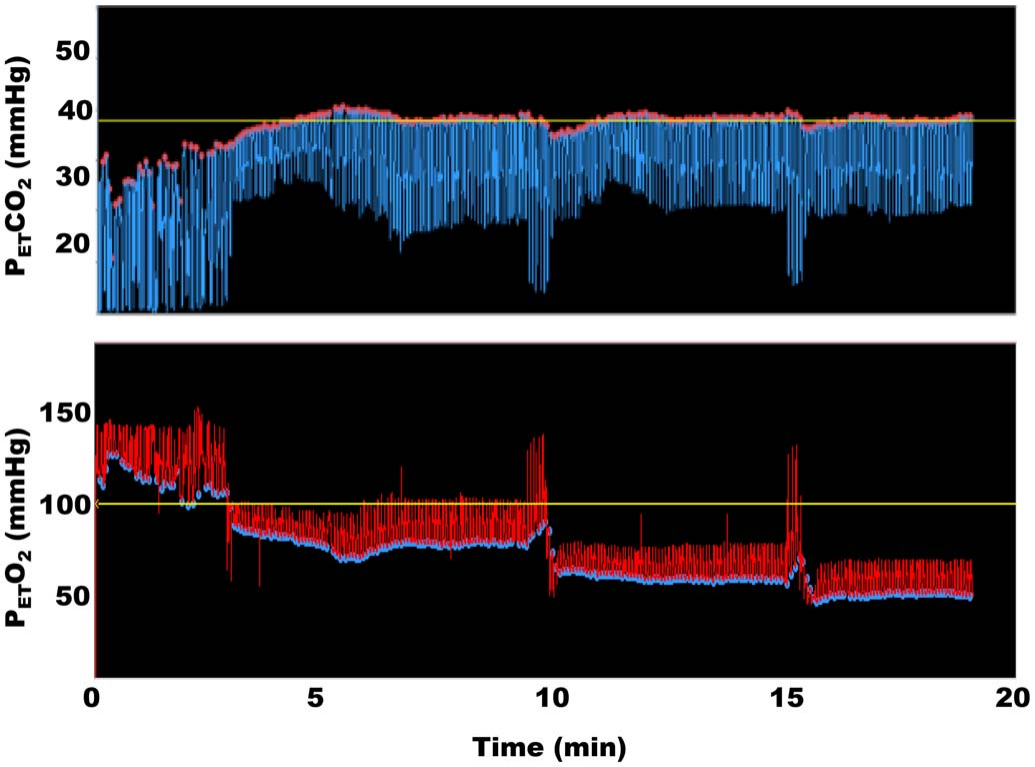
As in Figure 1 for the same subject but for the hypoxic end-tidal sequences. These data were obtained after two of the gas cylinders supplying the MPET were changed from a 10% oxygen mixture to a 6% mixture.

**Figure 3:**
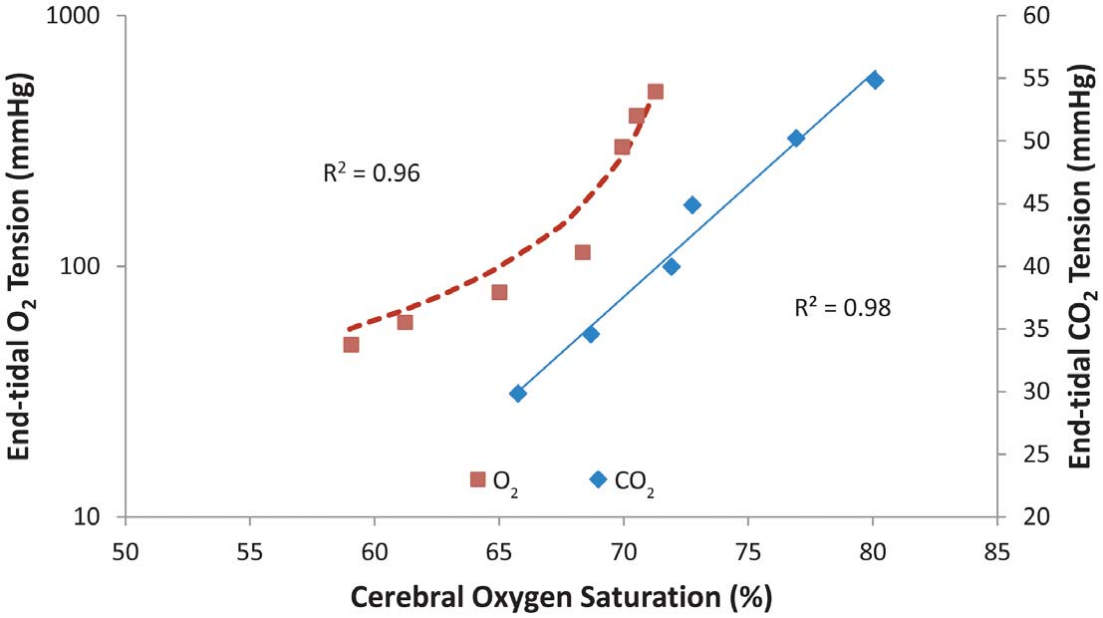
A study in one subject. The relationship between end-tidal carbon dioxide and cerebral saturation demonstrating the linear relationship between the two variables (individual points in blue diamonds). The curve fit the equation y = 2.16×−112. The linear curve fit for these data was R^2^ = 0.98. The relationship between end-tidal oxygen and cerebral saturation demonstrating the log-linear relationship between the two variables (individual points in red squares). The hyperbolic curve fit for these data was R^2^ = 0.96. The curve fit the equation y = b/(x−a): where b = −774 and a = 72.8; the asymptote for maximal saturation for hyperoxia.

Thirteen studies were undertaken. The first subject was excluded as end-tidal gas targeting was not adequate with a series of missed or poorly realized targets. Data are reported for the other 12 studies, and the subject demographics are listed in Table 1. The mean±SD baseline end-tidal carbon dioxide and oxygen tensions were 37±2 mmHg and 110±4 mmHg respectively. The time course of the experimental target sequence for one subject is shown in Figure 1 and 2. The mean±SD changes in end-tidal carbon dioxide tensions under baseline isoxic conditions are presented in Table 2. End-tidal clamping of oxygen tension was within ±4 mmHg for the group as a whole. The end-tidal carbon dioxide targets were within ±2 mmHg of their desired values for all measurement periods, but only 4/12 subjects were able to achieve a 15 mmHg decrement from their baseline values. A significant increase in blood pressure and heart rate was seen with increases in carbon dioxide tension (group×time interactions for repeated measures ANOVA p<0.001 for both variables). The changes in end-tidal oxygen tensions under baseline isocapnic conditions are presented in Table 3. In 2/12 subjects end-tidal targeting to 500 mmHg was not achieved, and one subject did not effectively achieve a target end-tidal oxygen tension of 50 mmHg. Significant decreases in pulse oximetry (SpO_2_) were seen at end-tidal tensions of oxygen of 60 and 50 mmHg, (group×time interactions for repeated measures ANOVA p<0.001). An individual example of the changes in cerebral saturation under the study conditions is depicted in Figure 3. The relationship between cerebral saturation and end-tidal carbon dioxide is presented in Table 4 for the group data. The mean calculated change in cerebral saturation/mmHg change in carbon dioxide tension was 0.48±0.09 percent/mmHg increase in carbon dioxide tension. The relationship between end-tidal carbon dioxide concentrations and cerebral saturation was well described in all subjects by a linear relationship (mean±SD R^2^-value 0.92±0.06). The relationship between end-tidal oxygen concentration and cerebral saturation was best graphed on a log-linear scale and in most cases best fit by a rectangular hyperbolic function (mean R^2^-value 0.85±0.10). Because of the hyperbolic curve fit we additionally analyzed the mean calculated change in cerebral saturation/mmHg change in oxygen tension as two linear data sets: end-tidal oxygen tensions from 50 – 100 mmHg and 100–500 mmHg. For the hypoxic-normoxic range the mean calculated change in cerebral saturation was 0.14±0.04 percent/mmHg increase in oxygen tension. For the normoxic-hyperoxic range the change was 0.009±0.003 percent/mmHg increase in oxygen tension - only 7 percent of the response at the lower range of oxygen tensions.

**Table 1:** Demographics.

Gender	10 M/3 F
**Age**	29.4±4.4
**Weight**	70±7
**Height**	172±9
**ETO_2_**	110±4
**ETCO_2_**	37±2
**SpO_2_**	98±1
**HR**	77±11
**Systolic BP**	122±11
**Diastolic BP**	77±11

Age in years.

Weight in kg.

Height in cm.

ET in mmHg.

SpO2 in %.

HR in bpm.

BP in mmHg.

**Table 2:** Changes in ETCO2 with isoxia.

Sequence	ETCO_2_	ETO_2_	SPO_2_	HR	Sys BP	Dia BP
B/L	37±2	110±4	98±1	77±11	122±11	77±11
ETCO_2_ −5 mmHg	32±2	109±4	98±1	78±12	122±12	76±6
ETCO_2_ −10 mmHg	27±2	110±5	99±1	77±13	122±12	77±13
ETCO_2_ −15 mmHg	37±2	110±4	98±1	77±11	122±11	77±11
ETCO_2_ +5 mmHg	42±2	110±4	98±1	75±11	125±11	79±6
ETCO_2_ +10 mmHg	47±2	110±4	98±1	79±10	128±11*	81±7*
ETCO_2_ +15 mmHg	52±2	110±4	99±1	82±12*	135±11*	82±12*

Where:

B/L is baseline.

* p<0.05 Tukey’s test vs. B/L.

**Table 3:** Changes in ETO2 with isocapnia.

Sequence	ETCO_2_	ETO_2_	SPO_2_	HR	Sys BP	Dia BP
B/L	37±2	110±4	98±1	77±11	122±11	77±11
ETO_2_ 300 mmHg	37±2	301±3	100±1*	73±10*	124±11	79±5
ETO_2_ 400 mmHg	37±2	400±2	100±1*	74±10*	124±9	73±10
ETO_2_ 500 mmHg	37±2	501±4	100±1*	73±11*	121±10	73±10
ETO_2_ 80 mmHg	37±2	79±1	96±2*	75±11	122±12	75±12
ETO_2_ 60 mmHg	37±2	59±1	92±2*	77±12	123±10	77±13
ETO_2_ 50 mmHg	37±2	49±1	85±1*	84±11*	118±13	84±11

Where:

B/L is baseline.

p<0.05 Tukey’s test vs. B/L.

**Table 4:** Linear Curve Fits for ETCO2 tensions and Cerebral Saturation.

Subject	m	b	R^2^	peak	CO_2_	nadir	CO_2_
1	2.75	−150	0.94	73	52	65	30
2	2.25	−114	0.79	75	55	64	24
3	1.83	−93	0.88	77	50	66	24
4	1.79	−84	0.98	80	55	66	30
5	2.32	−119	0.95	75	55	64	29
6	2.14	−117	0.92	80	54	67	27
7	1.47	−67	0.92	80	50	65	26
8	2.68	−160	0.91	80	53	69	24
9	2.02	−93	0.93	73	52	60	27
10	2.23	−120	0.94	78	51	65	21
11	1.94	−96	0.84	77	51	61	21
12	2.16	−112	0.98	75	50	64	25
**Mean**	**2.13**	−**110**	**0.92**	**77**	**52**	**65**	**26**
**SD**	**0.36**	**26**	**0.06**	**3**	**2**	**2**	**3**

Where:

y = mx+b.

The mean±SD maximal observed cerebral oxygen saturation (77±3 percent) occurred with hypercapnia (baseline values +15 mmHg; 52±2 mmHg) under isoxic conditions. This value exceeded the mean maximal cerebral saturation (73±3 percent; p = 0.00004 by paired t-test) seen with hyperoxia (mean value 410±98 mmHg) under isocapnic conditions. The cerebral saturation with hypercapnia was not significantly different from the calculated asymptote when the end-tidal oxygen to cerebral saturation relation was fitted with a rectangular hyperbolic function (77±3 *versus* 76±3 percent respectively; p = 0.068).

Cerebral saturation reached minimal values with a targeted end-tidal oxygen tension of 50 mmHg (61±3 percent) with isocapnia. The nadir for cerebral saturation under isoxic conditions at the lowest end-tidal carbon dioxide tensions achieved for each patient was 65±2 percent at a mean end-tidal carbon dioxide tension of 26±3 mmHg. This value did not differ significantly from the saturation (64±3 percent) seen at a mean isocapnic end-tidal oxygen tension of 59±1 mmHg; p = 0.58, paired t-test.

## Discussion

This study identifies important relationships between cerebral saturation and end-tidal gas concentrations in healthy subjects under the tightly controlled conditions permitted by the use of an MPET approach. There was a very reproducible output from the cerebral oximeter. Cerebral saturation varied in a linear fashion with changes in carbon dioxide tension under isoxic conditions. In contrast, cerebral saturation varied in a log-linear (rectangular hyperbolic) manner with changes in end-tidal oxygen tensions under isocapnic conditions. Hypocapnia consistently decreased cerebral saturation and hypercapnia increased it. Hypoxia in the range of 60 mmHg end-tidal tensions of oxygen under isocapnic conditions resulted in similar cerebral saturation as that seen with hypocapnia of 10–15 mmHg below baseline under isoxic conditions. With hypercapnia of 15 mmHg above baseline under isoxic conditions the greatest cerebral saturations were seen. Due to the hyperbolic nature of the end-tidal oxygen to cerebral saturation curve, it is evident that little increment in cerebral saturation is achieved by increasing inspired oxygen tension above 300 mmHg. These results have been obtained from a study of healthy young adults. Further studies are required to determine how they translate to various pathological conditions.

We noted progressively larger decreases in cerebral saturation as the end-tidal concentration of oxygen decreased, due to the hyperbolic nature of the correlation; a consequence, in part, of the sigmoidal curvature of the oxyhemoglobin saturation curve. The linear decrement in cerebral saturation with decreases in carbon dioxide tension under baseline isoxic conditions functions independently to decrease cerebral saturation. This suggests that in real life situations, with ascent to higher altitudes, a combination of the two effects could ensue as hypoxia activates peripheral and central chemoreceptors to increase minute ventilation, thereby simultaneously decreasing end-tidal carbon dioxide tension with increasing hypoxia. The nature of the combined relationship has not been examined here (as to whether or not the effects are additive or synergistic to decrease cerebral oxygen saturation).

Importantly the findings discussed here are contingent on the accuracy and precision of output by the cerebral oximeter. Accuracy and precision of measurement with cerebral oximeters is an ongoing debate. [12]–[16] We acknowledge that the evidence indicating what NIRS is really measuring is scarce and as a result our findings need be interpreted with caution. Absolute cerebral oximetry using laser-based optodes is claimed by the manufacturer of the Fore-Sight monitor which uses an algorithm based on 30∶70 percent arterial:venous oxygen saturation to reflect cerebral saturation. Less extracranial contamination has been demonstrated with this laser-based monitor compared to another more widely used monitor. [17] Good correlations between jugular venous oxygen saturation to cerebral saturation have been seen in clinical studies and use of laser-based optodes coupled with ultrasonograpy have been used as a trending monitor for an index of cerebral blood flow. [11] [18]–[19] The reproducibility of the results in the current study with very tightly controlled end-tidal gas concentrations suggest a consistency of measurement and given the significant cerebral blood flow differences that would accompany similar cerebral oxygen saturations with normocapnic hypoxia versus hypocapnic normoxia a common tissue measure is suggested. Extracranial tissue contamination with hypoxia can be easily envisioned to potentially degrade the cerebral saturation signal but not so with hypocapnic normoxia as extracranial tissue does not respond with nearly the same vasoconstrictive response as cerebral tissue to account for the desaturation observed with hypocapnia. [20] Also supportive of our findings is work by Mardimae et al. [21] demonstrating a hyperbolic cerebral blood flow response to alterations in oxygen tension as assessed by Doppler flowmetry.

These findings, cautiously interpreted, can provide some insight into management issues in neurocritical care and during neurosurgery. Brain oxygen saturation appears to benefit only minimally from end-tidal concentrations of oxygen above 300 mmHg – a consequence of the rectangular hyperbolic nature of the cerebral saturation response curve for increasing oxygen tensions (again, in part, a consequence of the sigmoidal oxyhemoglobin saturation curve; a consequence of the relationship of arterial blood oxygen tension and content – once the hemoglobin is fully saturated, higher oxygen tension only adds small volumes of oxygen dissolved in the plasma). Of note, in Table 5, the highest cerebral saturation with normocapnia was seen with the targeted end-tidal oxygen tension of 300 mmHg in 5/12 subjects. Higher inspired oxygen tension has been identified with decreased cerebral blood flow in some brain studies. [22]–[23] Perhaps a more representative effect of hyperoxia on cerebral saturation may be indicated by the asymptote for saturation – here 76±3 percent. Indeed, previous positron emission tomographic (PET) studies appear to corroborate our observations in neurocritical care patients. In these studies, increasing inspired oxygen from an F_I_O_2_ of 0.21 (PaO_2_ 99±23 mmHg) to F_I_O_2_ of 0.35–0.50 (PaO_2_ 226±68 mmHg) resulted in modest increases in cerebral tissue oxygen tensions (from 28±21 to 57±47 mmHg). [24] These changes in F_I_O_2_ would produce changes in cerebral saturation similar to our increases in end-tidal oxygen tension from baseline up to 300 mmHg – where an effect of increasing oxygen tension benefits cerebral saturation. Also noted in the PET study was an increase in cerebral metabolic rate for oxygen in ‘at risk’ tissue, and modest improvements in lactate/pyruvate ratio were also seen where tissue was ‘at risk’. Nevertheless, higher oxygen tensions were not examined in this study.

**Table 5:** Hyperbolic Curve Fits for ETO2 tensions and Cerebral Saturation.

Subject	asym	b	R^2^	peak	O_2_	nadir	O_2_
1	73	−1058	0.89	71	502	59	49
2	73	−3069	0.78	68	306	57	48
3	78	−1169	0.70	75	302	63	50
4	77	−814	0.81	75	398	66	59
5	75	−967	0.93	73	500	59	50
6	82	−1414	0.65	78	301	64	49
7	74	−888	0.95	73	500	60	50
8	80	−603	0.89	78	304	66	50
9	72	−1046	0.97	70	499	57	50
10	75	−602	0.82	73	306	62	50
11	76	−1358	0.79	74	499	65	48
12	73	−774	0.96	71	497	59	49
**Mean**	**76**	−**1147**	**0.85**	**73**	**410**	**61**	**50**
**SD**	**3**	**659**	**0.10**	**3**	**98**	**3**	**3**

Where:

y = b/(x−a).

a = asym.

The same group examined the effects of hypocapnia on injured brain with PET imaging. [25] A decrement in PaCO_2_ from 36±3 to 29±2 mmHg (equivalent to a decrease in carbon dioxide of 5–10 mmHg from baseline in our study) resulted in a significant reduction in cerebral blood flow, increased oxygen extraction ratio and ischemic brain volume. The authors suggested that moderate hypocapnia in an injured brain represents a significant ‘physiological challenge’ to the brain. Of particular note in this study was that the regional ischemic changes occurred without evidence of significant alterations is jugular venous oxygen saturation. Other work indicates that both hypercapnia and hypocapnia are associated with a worsened outcome in intubated but not spontaneously breathing head injured patients. [26] Improved survival was noted in patients with arterial carbon dioxide tensions between 30 and 49 mmHg in a total of 890 intubated and 2914 non intubated patients. [27] However, the range with good outcome when carbon dioxide tension was 40–49 mmHg is above what most clinicians would consider optimal for patients with head injury. A possible explanation for this counterintuitive finding is offered by our results, which indicate improved cerebral oxygenation at higher carbon dioxide tensions. In fact, in our healthy subjects hypercapnia was customarily associated with the highest cerebral saturations seen. It seems safe to conclude from the above evidence that hypocapnia is problematic for cerebral oxygenation both in health and with head injury. But the potential for modest hypercapnia to improve cerebral oxygenation under carefully monitored circumstances is suggested. In fully monitored neurocritical care patients - including cerebral oximetry and extraventricular drainage devices (EVDs) to aid intracranial volume control, our results suggest that mild hypercapnia could be entertained if cerebral saturation is problematic. Incremental increases in carbon dioxide tension potentially could be better tolerated without the attendant consequences of high inspired concentrations of oxygen which is often a first line therapy for management of the patient with cerebral hypoxia. Such a means to improve cerebral saturation is a recommended approach for management of decreases in cerebral saturation during open heart surgery [28].

Our findings are also relevant to issues of using hyperoxia and hypercapnia in the calibration of magnetic resonance imaging blood oxygen level dependent (BOLD) signals. In some studies, hypercapnia is induced to maximize cerebral blood flow to minimize deoxyhemoglobin and maximize the BOLD signal to provide an estimate of ‘M’ – the theoretical maximal BOLD signal. [8] [29]–[30] Another approach is to maximize the oxygen content in the arterial blood to increase the venous oxygen content and thereby minimize the deoxyhemoglobin. [29] [31] Our study provides validation that both approaches can be used, but indicates that greater cerebral oxygenation is found with the hypercapnic approach.

A limitation of our study is that it was a physiologic investigation of the independent effects of carbon dioxide and oxygen on cerebral saturation in young healthy adults. The role of carbon dioxide and oxygen in the presence of neuronal or neurovascular pathology is speculative. However, the results we obtained seem reliable as they were very reproducible across the study population with all subjects demonstrating a clear linear relationship between end-tidal carbon dioxide tension and cerebral saturation and a log-linear hyperbolic relationship between end-tidal oxygen tension and cerebral saturation.

These findings suggest that a potential exists to use NIRS with independent end-tidal carbon dioxide and oxygen targeting to identify a patient’s carbon dioxide and oxygen response curves and optimize cerebral saturation. A further consideration is to employ the MPET approach to generate cerebrovascular reactivity (CVR) maps with concurrent BOLD-MRI scans, [32]–[33] as previously demonstrated, to regionally ‘tune’ each neurocritical care patient’s cerebral oxygenation.
